# Data on corrosion and scaling potential of drinking water resources using stability indices in Jolfa, East Azerbaijan, Iran

**DOI:** 10.1016/j.dib.2017.11.099

**Published:** 2017-12-06

**Authors:** Mahmood Yousefi, Hossein Najafi Saleh, Amir Hossein Mahvi, Mahmood Alimohammadi, Ramin Nabizadeh, Ali Akbar Mohammadi

**Affiliations:** a Students Research Committee, Neyshabur University of Medical Sciences, Neyshabur, Iran; b Torbat Heydariyeh University of Medical Sciences, Torbat Heydariyeh, Iran; c Department of Environmental Health Engineering, School of Public Health, Tehran University of Medical Science, Tehran, Iran; d Center for Solid Waste Research, Institute for Environmental Research, Tehran University of Medical Sciences, Tehran, Iran; e Center for Air Pollution Research, Institute for Environmental Research, Tehran University of Medical Sciences, Tehran, Iran; f Department of Environmental Health Engineering, Neyshabur University of Medical Sciences, Neyshabur, Iran

**Keywords:** Corrosion and scaling potential, Stability indices, Ground water, Jolfa

## Abstract

This cross-sectional study was conducted on the drinking water resources of the city of Jolfa (East Azerbaijan province, Iran) from samples taken from 30 wells. Calcium hardness, pH, total alkalinity, TDS, temperature and other chemical parameters were measured using standard methods. The Langelier, Rayzner, Puckhorius and aggressive indices were calculated. The results showed that the Langelier, Reynar, Puckorius, Larson-skold and aggressive indices were 1.15 (± 0.43), 6.92 (± 0.54), 6.42 (± 0.9), 0.85 (± 0.72) and 12.79 (± 0.47), respectively. In terms of water classification, 30% of samples fell into the NaCl category and 26.6% in the NaHCO_3_ category and 43.4% samples in the CaHCO_3_, MgHCO_3_ and MgCl category. The sedimentation indices indicated that the water of the wells could be considered as corrosive.

**Specifications Table**

**Table:** 

Subject area	Chemistry
More specific subject area	Describe narrower subject area
Type of data	Tables, Figure
How data was acquired	To calculate the corrosion indices, 120 water samples were collected, stored and transferred to the lab using standard methods and the water quality parameters such as temperature, electrical conductivity, total dissolved solids, pH, dissolved oxygen, calcium hardness, alkalinity, chloride and sulfate were measured. The gravimetric method was used to measure the dissolved solids and the titration method was used to determine alkalinity. Sulfate ions were measured based on turbidity measurement at 420 nm using a DR5000 spectrophotometer. Residual chlorine and pH measurement was carried out using test kits and water temperature was measured with a thermometer at the sampling points
Data format	Raw, Analyzed
Experimental factors	The mentioned parameters above, in abstract section, were analyzed according to the standards for water and wastewater treatment handbook.
Experimental features	The levels of physical and chemical parameters were determined.
Data source location	Jolfa, East Azerbaijan province, Iran
Data accessibility	The data are available whit this article

**Value of the data**

• Calculation of corrosion indices showed that the chemical quality of the water was imbalanced and could cause corrosion to the water system and other facilities.
• The water quality and the potential for corrosion in all distribution systems is necessary to avoid economic loss and avert adverse effects on health.
• Comparison of five stability indices showed that water conditions in all parts of this study are supersaturated.

## 1 Data

The data presented here deals with monitoring of the physical and chemical properties of pH, EC, TDS, HCO_3_^−^, CO_3_^−^, SO_4_^2−^, CL^−^, Ca^2 +^ , Mg^2 +^ and Na ^+^ as shown in Table 2, Table 3, respectively. The results of the calculations for the Langelier, Ryzener, Puckorius, Aggressive and Larson indices are presented for Jolfa in Table 4. All indices other than the AI index indicated that the water is corrosive. The Langelier index was greater than zero in 90% of samples. Based on the average of this index, the water can be classified as supersaturated; thus, according to the Langelier index, the water is not corrosive. In all samples, (60%) the Ryzener index was between 6 and 7 and it can be concluded that the water samples are saturated (Table 4). The water samples were classified as 30% in the NaCl category, 26.6% in the NaHCO_3_ category and 43.4% in the CaHCO_3_, MgHCO_3_ and MgCl category (Table 5).

**Table 1:** Summary of water stability indices in present study [1], [2], [3], [4].

	Equation			Index value	Water condition		
Langelier saturation	LSI = pH−pHs			LSI > 0	Super saturated, tend to precipitate CaCO_3_		
index (LSI)	pHs = A + B − log (Ca^2 +^ )− log			LSI = 0	Saturated, CaCO_3_ is in equilibrium		
	(Alk) pH < = 9.3						
	pHs = (9.3 + A + B) − (C + D)			LSI < 0	Under saturated, tend to dissolve solid CaCO_3_		
	(3) pH > 9.3						
			
Ryznar stabilityindex (RSI)	RSI = 2pHs−pH			RSI < 6	Super saturated, tend to precipitate CaCO_3_		
				6 < RSI < 7	Saturated, CaCO_3_ is in equilibrium		
				RSI > 7	Under saturated, tend to dissolve solidCaCO_3_		
			
Puckorius scalingindex (PSI)	PSI = 2 (pHeq)−pHs			PSI < 6	Scaling is unlikely to occur		
	pH = 1.465 + log			PSI > 7	Likely to dissolve scale		
	(T.ALK) + 4.54						
	pHeq = 1.465×log(T.ALK) + 4.54						
			
Larson-skold index(LS)	Ls = (Cl^−^ + SO_4_^2−^)/(HCO_3_^−^ +			LS < 0.8	Chloride and sulfate are unlikely to interfere with the		
	CO_3_^2−^)				formation of protecting film		
				0.8 < LS < 1.2	Corrosion rates may be higher than expected		
				LS > 1.2	High rates of localized corrosion may be expected		
			
Aggressive index	AI = pH + log[(Alk)(H)]			AI > 12	Non aggressive		
(AI)				10 < AI < 12	Moderately aggressive		
				AI < 10	Very aggressive

**Table 2:** Physical and chemical characteristics of water quality of distribution networks of Jolfa city.

**Number**	**Ca**^**2 +**^	**Mg**^**2 +**^	**Na**^**+**^	**K**^**+**^	**CO**_**3**_^**2−**^	**HCO**_**3**_^**−**^	**TH**
**Well**	(mg/l)	(mg/l)	(mg/l)	(mg/l)	(mg/l)	(mg/l)	As CaCO_3_ (mg/l)
W1	144.00	87.84	349.6	7.41	0	600.85	721.29
W2	25.60	25.864	64.4	1.17	6	317.2	170.43
W3	44.00	35.624	45.54	2.73	6	314.15	256.57
W4	54.40	33.672	50.6	1.56	0	335.5	274.5
W5	18.40	25.864	167.9	3.12	15	381.25	152.45
W6	72.00	55.144	170.2	7.8	12	488	406.87
W7	67.20	35.624	184	3.12	0	448.35	314.5
W8	176.00	129.32	483	7.41	0	506.3	972.01
W9	66.00	18.91	33.12	1.95	0	219.6	242.67
W10	70.00	17.69	30.82	1.95	0	219.6	247.64
W11	61.40	22.57	11.96	2.34	28.8	190.32	246.26
W12	69.00	22.204	28.75	1.56	0	225.7	263.73
W13	160.00	97.6	349.6	7.41	0	649.65	801.44
W14	44.00	75.64	188.6	4.29	15	298.9	421.35
W15	120.00	87.84	181.7	7.02	0	741.15	661.37
W16	60.00	39.04	381.8	2.73	0	454.45	310.59
W17	72.00	56.12	170.2	7.8	12	488	410.89
W18	88.00	107.36	200.1	7.02	0	585.6	661.84
W19	52.00	39.04	31.28	1.17	0	366	290.61
W20	132.00	163.48	310.5	3.12	0	527.65	1002.81
W21	27.20	30.256	14.95	1.17	15	179.95	192.51
W22	176.00	122	471.5	7.41	0	439.2	941.87
W23	160.00	97.6	345	7.41	0	649.65	801.44
W24	180.00	85.4	126.5	5.46	0	747.25	801.14
W25	52.00	39.04	31.28	1.17	0	366	290.61
W26	132.00	168.36	310.5	3.12	0	527.65	1022.91
W27	18.40	25.864	163.3	3.12	0	408.7	152.45
W28	63.20	56.12	165.6	3.9	0	405.65	388.91
W29	160.00	97.6	345	7.41	0	649.65	801.44
W30	180.00	85.4	115	5.46	0	716.75	801.14
Mean	91.49	66.14	184.08	4.28	3.66	448.29	500.81
Max	180	168.36	483	7.8	28.8	747.25	1022.91
Min	18.4	17.69	11.96	1.17	0	179.95	152.45
S.D	55.31	45.48	148.38	2.52	8.09	174.63	300.55

**Table 3:** Physical and chemical characteristics of water quality of distribution networks of Jolfa city.

**Number**	**ALK**	**CL**^**−**^	**SO**_**4**_^**2−**^	**EC**	**TDS**	**pH**	**HCO**_**3**_^**−**^	**CaH**
**Well**	as CaCO_3_ (mg/l)	(mg/l)	(mg/l)	(μmhos/cm)	(mg/l)		(mg/l)	as CaCO_3_ (mg/l)
W1	600.85	532.5	235.2	3060	1788	8.2	600.85	360
W2	323.20	24.85	4.8	663	374.4	8.7	317.2	64
W3	320.15	28.4	48	654	430.8	8.7	314.15	110
W4	335.50	42.6	48	573	465	8.1	335.5	136
W5	396.25	60.35	96	1092	627.6	9	381.25	46
W6	500.00	152.65	144	3330	943.8	8.5	488	180
W7	448.35	184.6	86.4	636	863.4	8.1	448.35	168
W8	506.30	754.375	528	7080	2436	8.2	506.3	440
W9	219.60	69.225	36	620	403	7.75	219.6	165
W10	219.60	69.58	35.52	620	403	7.75	219.6	175
W11	219.12	18.46	45.12	574	340	8.37	190.32	153.5
W12	225.70	69.935	38.4	640	416	7.8	225.7	172.5
W13	649.65	532.5	273.6	3140	1884	7.4	649.65	400
W14	313.90	213	254.4	1673	1003.8	8.6	298.9	110
W15	741.15	230.75	124.8	2130	1278	7.5	741.15	300
W16	454.45	443.75	139.2	2290	1374	7.9	454.45	150
W17	500.00	156.2	144	1582	949.2	8.5	488	180
W18	585.60	399.375	57.6	2210	1326	7.7	585.6	220
W19	366.00	23.075	24	720	432	7.2	366	130
W20	527.65	621.25	355.2	3360	2016	7.7	527.65	330
W21	194.95	12.425	33.6	454	272.4	8.6	179.95	68
W22	439.20	754.375	528	3950	2370	7.9	439.2	440
W23	649.65	532.5	264	3120	1872	7	649.65	400
W24	747.25	227.2	144	2170	1302	7	747.25	450
W25	366.00	23.075	24	720	432	7.1	366	130
W26	527.65	621.25	374.4	3400	2040	7.5	527.65	330
W27	408.70	53.25	96	1024	614.4	7.9	408.7	46
W28	405.65	106.5	259.2	1509	905.4	7.5	405.65	158
W29	649.65	532.5	264	3120	1872	7	649.65	400
W30	716.75	227.2	144	2120	1272	7.5	716.75	450
Mean	451.95	257.26	161.65	1941.13	1090.2	7.89	448.29	228.73
Max	747.25	754.38	528	7080	2436	9	747.25	450
Min	194.95	12.43	4.8	454	272.4	7	179.95	46
S.D	175.43	248.71	151.68	1691.65	699.97	0.59	174.63	138.28

**Table 4:** Results of Water stability indices calculations samples obtained from Jolfa city.

**Number Well**	**Index**				
	**LSI**	**RSI**	**PSI**	**LS**	**AI**
W1	1.13	5.93	5.52	1.28	13.54
W2	0.82	7.05	7.54	0.09	13.02
W3	1.06	6.59	7.08	0.24	13.25
W4	0.58	6.94	6.80	0.27	12.76
W5	1.01	6.98	7.63	0.39	13.26
W6	1.04	6.42	6.43	0.59	13.45
W7	0.79	6.52	6.20	0.60	12.98
W8	1.01	6.18	5.88	2.53	13.55
W9	0.12	7.50	7.28	0.48	12.31
W10	0.15	7.45	7.23	0.48	12.33
W11	0.72	6.93	7.33	0.29	12.90
W12	0.20	7.40	7.21	0.48	12.39
W13	0.41	6.58	5.32	1.24	12.81
W14	0.83	6.94	7.34	1.49	13.14
W15	0.50	6.49	5.25	0.48	12.85
W16	0.38	7.14	6.61	1.28	12.73
W17	1.15	6.19	6.20	0.60	13.45
W18	0.46	6.78	5.88	0.78	12.81
W19	-0.32	7.85	6.75	0.13	11.88
W20	0.52	6.65	5.82	1.85	12.94
W21	0.57	7.46	8.17	0.24	12.72
W22	0.74	6.41	5.90	2.92	13.19
W23	0.01	6.98	5.32	1.23	12.41
W24	0.18	6.64	4.89	0.50	12.53
W25	-0.42	7.95	6.75	0.13	11.78
W26	0.32	6.85	5.83	1.89	12.74
W27	-0.07	8.04	7.57	0.37	12.17
W28	0.01	7.47	6.61	0.90	12.31
W29	0.01	6.98	5.32	1.23	12.41
W30	0.67	6.17	4.95	0.52	13.01
Mean	0.49	6.92	6.42	0.85	12.79
Max	1.15	6.92	6.42	0.85	12.79
Min	-0.42	5.93	4.89	0.09	11.78
S.D	0.43	0.54	0.9	0.72	0.47

**Table 5:** Water quality classification for individual samples.

Number Well	Water categories based on TDS	Water category based on Piper chart	
W1	Brackish water	Na ^+^	Cl^−^
W2	Fresh water	Na ^+^	HCO_3_^−^
W3	Fresh water	Mg^2 +^	HCO_3_^−^
W4	Fresh water	Mg^2 +^	HCO_3_^−^
W5	Fresh water	Na ^+^	HCO_3_^−^
W6	Fresh water	Na ^+^	HCO_3_^−^
W7	Fresh water	Na ^+^	HCO_3_^−^
W8	Brackish water	Na ^+^	Cl^−^
W9	Fresh water	Ca^2 +^	HCO_3_^−^
W10	Fresh water	Ca^2 +^	HCO_3_^−^
W11	Fresh water	Ca^2 +^	HCO_3_^−^
W12	Fresh water	Ca^2 +^	HCO_3_^−^
W13	Brackish water	Na ^+^	Cl^−^
W14	Brackish water	Na ^+^	Cl^−^
W15	Brackish water	Na ^+^	HCO_3_^−^
W16	Brackish water	Na ^+^	Cl^−^
W17	Fresh water	Na ^+^	HCO_3_^−^
W18	Brackish water	Mg^2 +^	Cl^−^
W19	Fresh water	Mg^2 +^	HCO_3_^−^
W20	Brackish water	Na ^+^	Cl^−^
W21	Fresh water	Mg^2 +^	HCO_3_^−^
W22	Brackish water	Na ^+^	Cl^−^
W23	Brackish water	Na ^+^	Cl^−^
W24	Brackish water	Ca^2 +^	HCO_3_^−^
W25	Fresh water	Mg^2 +^	HCO_3_^−^
W26	Brackish water	Mg^2 +^	Cl^−^
W27	Fresh water	Na ^+^	HCO_3_^−^
W28	Fresh water	Na ^+^	HCO_3_^−^
W29	Brackish water	Na ^+^	Cl^−^
W30	Brackish water	Ca^2 +^	HCO_3_^−^

## 2 Experimental design, materials and methods

### 2.1 Study area description

**Fig. 1:**
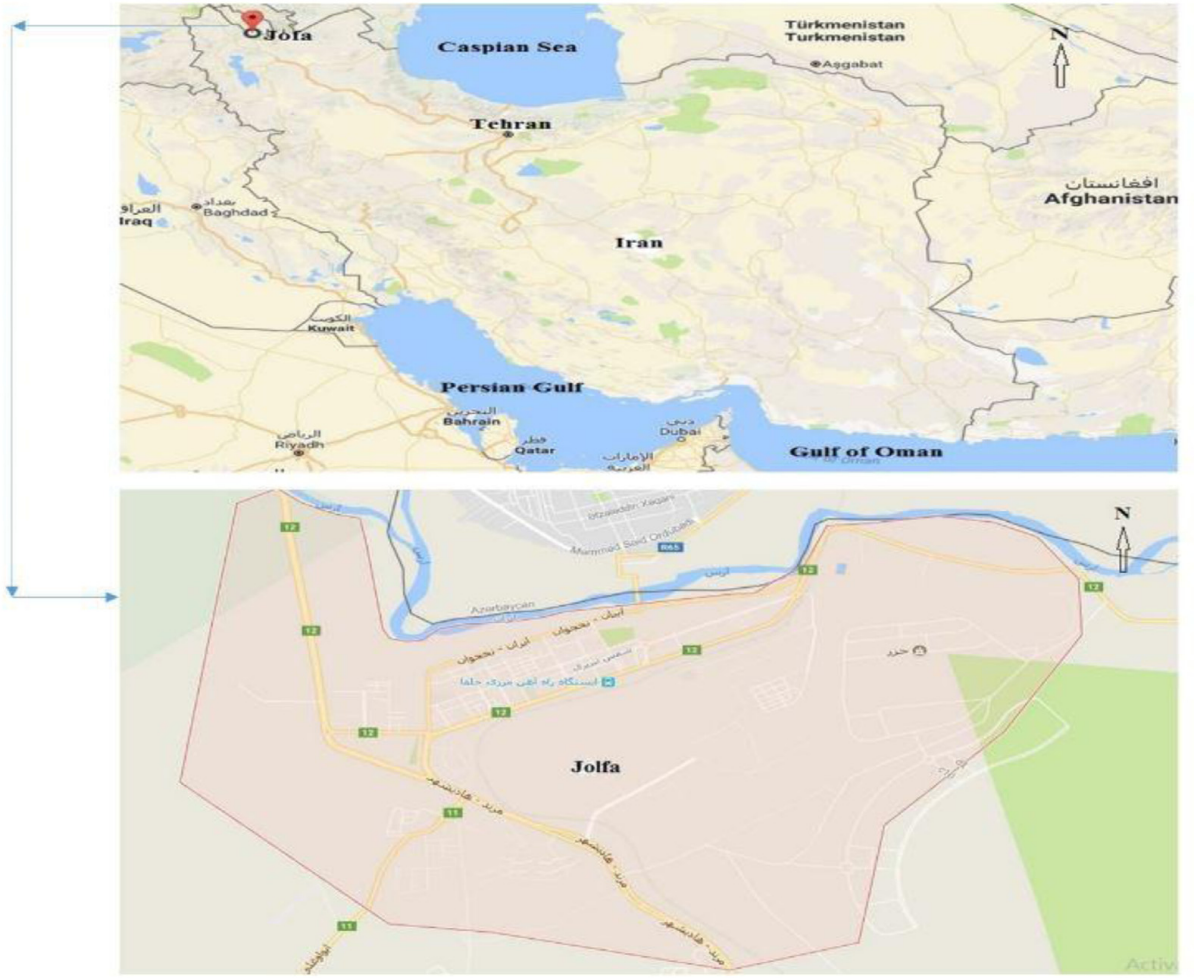
Location of the study area in Jolfa city, East Azerbaijan, Iran.

Jolfa is the capital of Jolfa county in East Azerbaijan province in Iran. Jolfa county is located in northern East Azerbaijan province at UTM coordinates of X = 45.17 to 46.31 east longitude and Y = 38.39 to 39.2 north latitude. The city borders the river Aras and the autonomous republic of Nakhchivan and the Republic of Armenia and Azerbaijan to the north [Fig. 1].

### 2.2 Sample collection and analytical procedures

To calculate the corrosion indices, 120 water samples were collected, stored and transferred to the lab using standard methods and the water quality parameters such as temperature, electrical conductivity, total dissolved solids, pH, dissolved oxygen, calcium hardness, alkalinity, chloride and sulfate were measured. The gravimetric method was used to measure the dissolved solids and the titration method was used to determine alkalinity. Sulfate ions were measured based on turbidity measurement at 420 nm using a DR5000 spectrophotometer. Residual chlorine and pH measurement was carried out using test kits and water temperature was measured with a thermometer at the sampling points [5], [6], [7], [8], [9], [10], [11]. The equations of the corrosion indices and their interpretations are summarized in Table 1.
